# Imaging assessment of mitral and aortic regurgitation: current state of the art

**DOI:** 10.1136/heartjnl-2019-316216

**Published:** 2020-08-17

**Authors:** Richard Paul Steeds, Saul G Myerson

**Affiliations:** 1 Department of Cardiology, Queen Elizabeth Hospital, Birmingham, UK; 2 Honorary Reader, Institute of Cardiovascular Sciences, University of Birmingham, Birmingham, UK; 3 Cardiovascular Medicine, University of Oxford, Oxford, UK

**Keywords:** mitral regurgitation, aortic regurgitation, valvular heart disease, echocardiography, cardiac magnetic resonance (CMR) imaging

**Learning objectives**

- To understand the advantages and disadvantages of imaging techniques in identifying aetiology and mechanisms of mitral and aortic regurgitation.
- To understand the key echocardiographic methods of quantifying severity of mitral regurgitation (MR) and aortic regurgitation (AR), and their advantages and limitations.
- To be aware of the relative advantages and disadvantages of cardiac magnetic resonance in quantifying severity of MR and AR.

## Introduction

Valvular heart disease (VHD) affects 1 in 50 of the general population and 1 in 3 of the over 65s, and is associated with increased morbidity and premature mortality.^1^ Among adults presenting to hospital with severe VHD, mitral regurgitation (MR: 21.3%) is the second and aortic regurgitation (AR: 5.3%) is the third most common valve lesion after aortic stenosis (41.2%).^2^ The predominance of MR above AR is also found in community-based studies, although it is much more common to find mild and moderate VHDs than severe disease in the general population.^1^ VHD of all types is more common with advancing age, and given the steady rise of life expectancy in the Western world, the frequency of patients presenting with MR and AR is increasing. Despite clear evidence of benefit from surgery when performed in a timely fashion, many patients undergo operation late in the course of disease, with more advanced symptoms and higher rates of heart failure and ventricular dysfunction.^2^ There is a long latent period in the majority of patients with either MR or AR, and timely diagnosis, careful monitoring and early referral remain critical to care.

## Haemodynamics and imaging of valvular regurgitation

The severity of regurgitation through the MV and AV is governed by the Gorlin formula, which states that flow through an orifice varies by the square root of the pressure gradient across the orifice, the duration of flow and a discharge coefficient.^3^ Therefore, the main determinants of the regurgitant volume (RVol) are (1) the size of the regurgitant orifice; (2) the pressure gradient between the left ventricle (LV) and the left atrium (LA) in MR, and between the aortic root and the LV in AR. In most patients, the size of the regurgitant orifice is not fixed but varies with dynamic changes in geometry, pressure and volume. For example, changes in volume status can have a significant impact on the assessment of MR, with reduction in severity following dialysis and after diuretic therapy compared with imaging assessment before treatment. Both the dynamic nature of the regurgitant orifice and the relationship between pressure and flow mean that images of the patient with regurgitation must be understood in the context of the haemodynamic status of the patient. Therefore, all reports of severity should include heart rate and blood pressure, while the use of sedation is an important consideration when comparing findings on transthoracic echocardiography (TTE) and transoesophageal echocardiography (TOE). Equally, it should be borne in mind that most assessments of MR and AR occur at rest, and there may be significant changes on exertion (either an increase or decrease in MR, depending on the mechanism; and a decrease in AR due to the shortening of diastole with higher heart rates). Another important factor in assessing regurgitation is the time course of onset: for example, in chronic MR, there is chamber dilatation, increased LV compliance and lower pulmonary venous pressure, whereas in acute MR, which is usually into a low-compliance LV, there is little cavity dilatation and high pulmonary venous pressure. In acute MR, this means the RVol may be much smaller than expected, and even a moderate degree may cause symptoms as the LV has not had time to accommodate for the MR.^4^

## Identifying the mechanism of pathological regurgitation

Access to echocardiography is critical for diagnosis of valve disease.^5^ TTE with colour Doppler remains the main modality for detection of regurgitation. Note that small degrees of physiological MR through a structurally normal valve will be detected in 10%–20% of children and closing volumes in a higher percentage.^6^ In contrast, physiological AR is rare, occurs in less than 1% of cases and should be considered pathological until proven otherwise.

**Figure 1:**
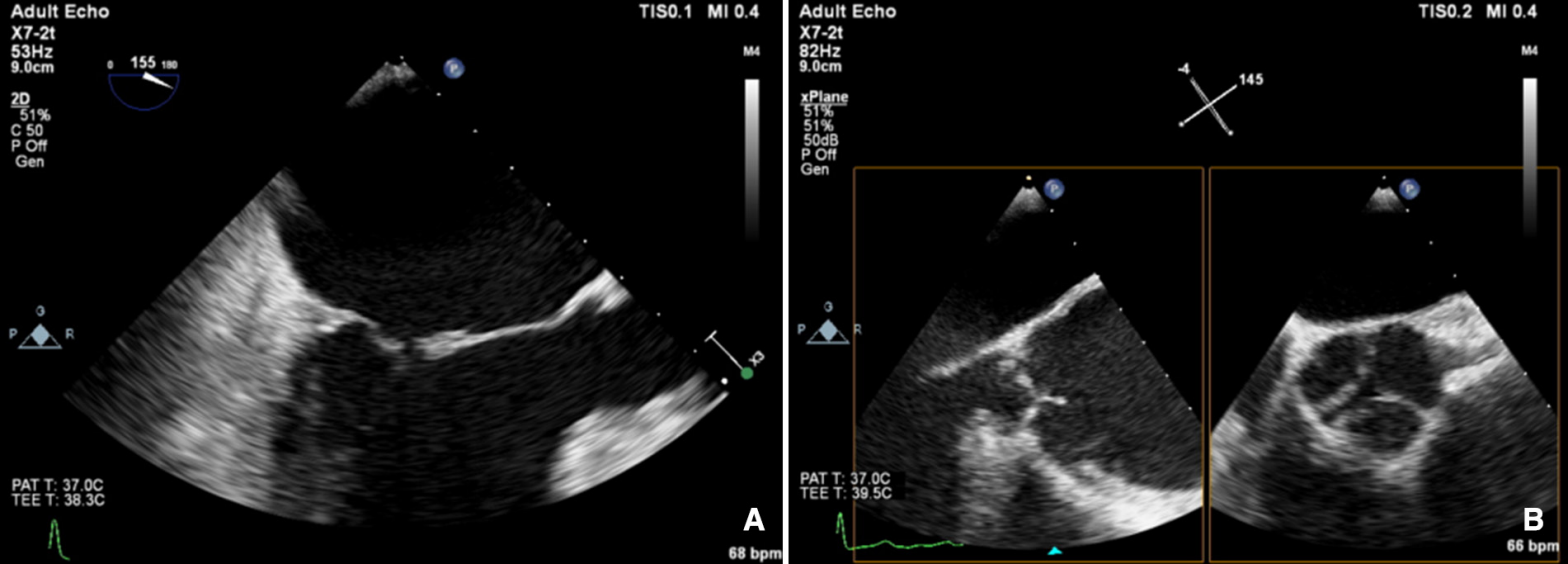
Carpentier type 1A, normal leaflet motion: annular dilatation (A) mitral regurgitation and (B) aortic regurgitation.

**Figure 2:**
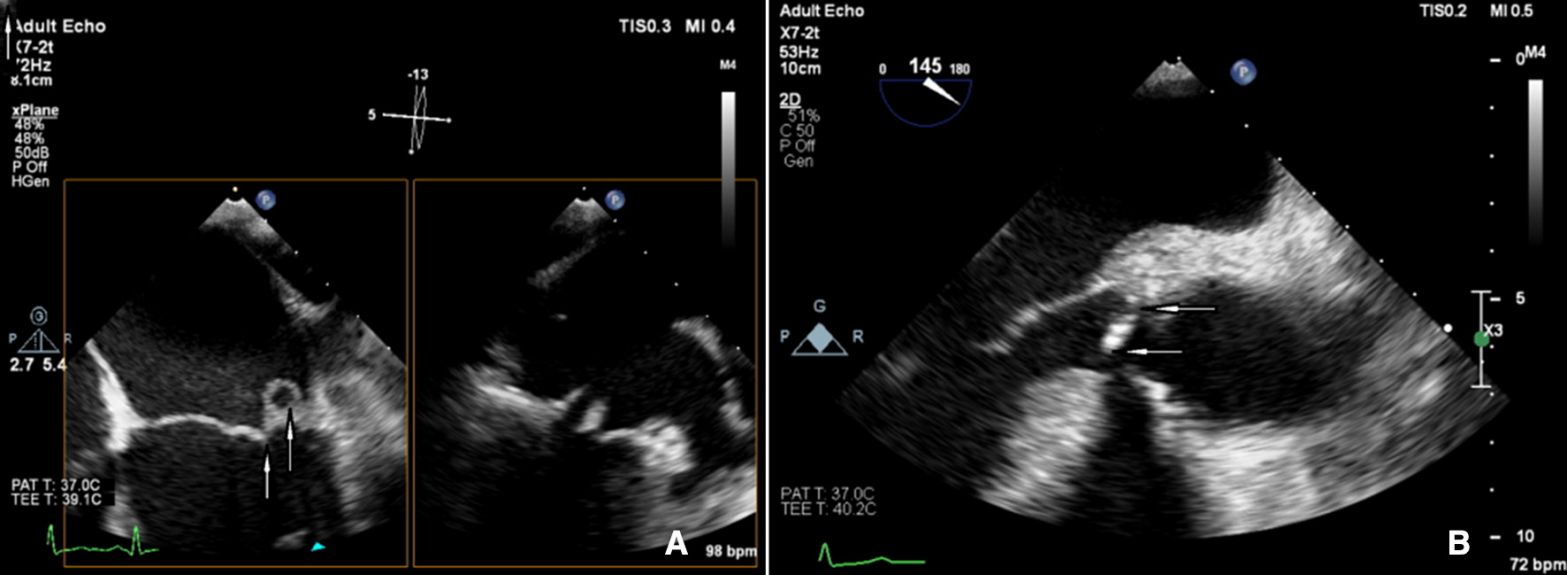
Carpentier type 1B, normal leaflet motion: leaflet perforation (A) mitral regurgitation and (B) aortic regurgitation.

**Figure 3:**
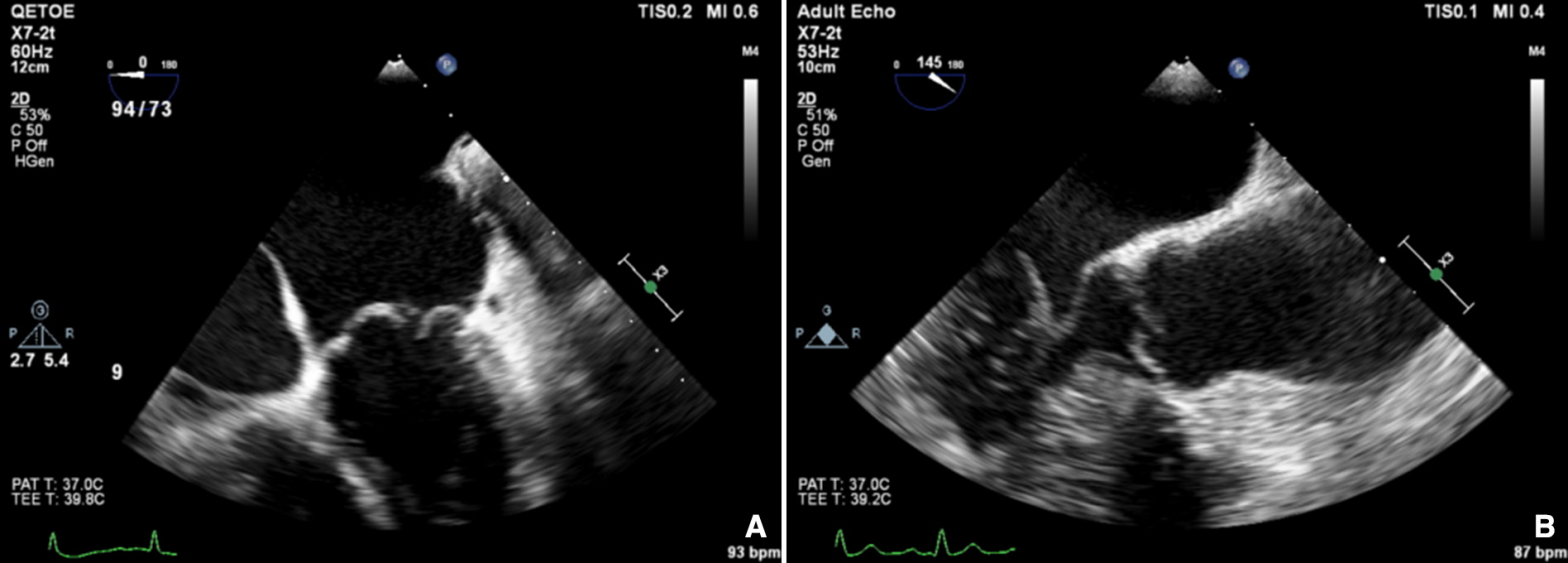
Carpentier type 2: excessive leaflet motion with prolapse of leaflet tip behind annulus (A) mitral regurgitation and (B) aortic regurgitation.

**Figure 4:**
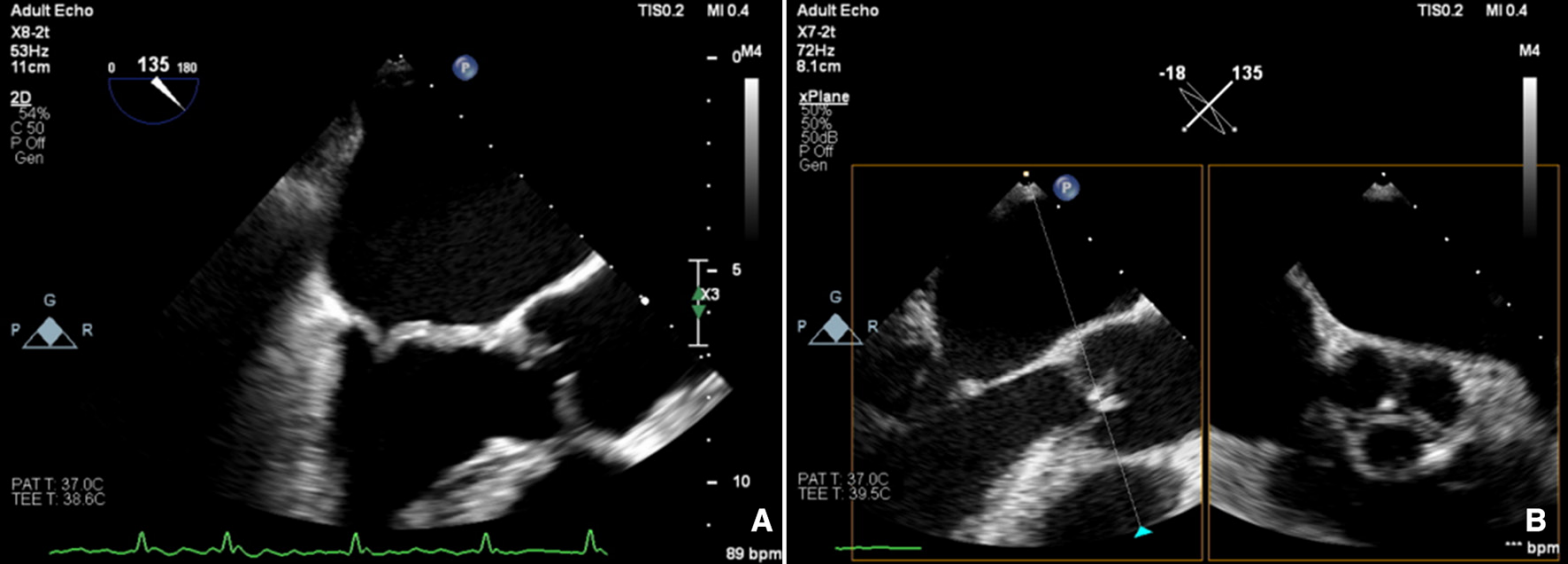
Carpentier type 3A: restrictive leaflet motion—systole and diastole: rheumatic (A) mitral regurgitation and (B) aortic regurgitation.

**Figure 5:**
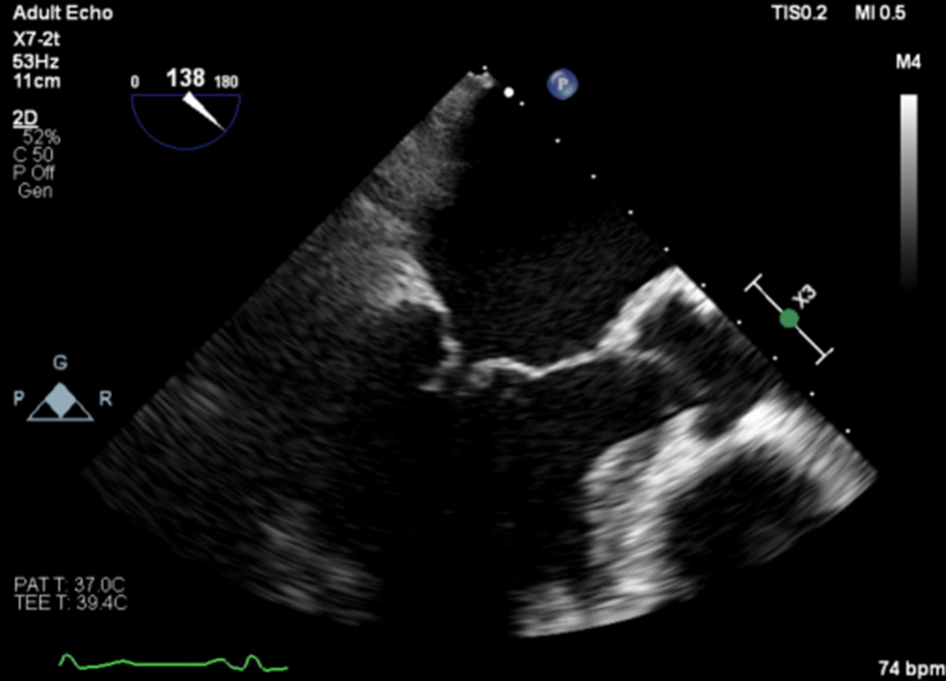
Carpentier type 3B: restrictive leaflet motion—systole (closure): ischaemic mitral regurgitation.

Once regurgitation has been detected, the key step is to determine the cause. This is particularly the case in the patient with MR, as the natural history, classification of severity and treatment choices all depend on whether regurgitation is ‘primary’, due to a fault in the valve apparatus, or ‘secondary’, due to disease of the LV (table 1). Most cases of AR, on the other hand, are secondary to an aortic syndrome, either as a result of atherosclerotic aortic root dilatation or as part of the aortopathy that can coexist in those with a bicuspid aortic valve^7^ (table 2). In both MR and AR, it is good practice to use the Carpentier classification to describe abnormalities of leaflet motion and thereby define mechanism (figures 1–5). This helps to identify the pathology, may guide the approach to surgery and can help to predict recurrence.^8^

**Table 1:** Causes of mitral regurgitation

Primary (valve apparatus)		Usual Carpentier mechanism
Congenital	Cleft	II
	Parachute	II
Myxomatous	Fibroelastic deficiency	II
	Barlow disease	II
Degenerative	Age-related	
	Mucopolysaccharidoses	
Inflammatory	Rheumatic	IIIA
	Radiation (lymphoma, breast)	IIIA
	Drugs (dopamine agonists)	IIIA
	Infective endocarditis	IB
	Collagen vascular diseases	
Secondary (ventricular/atrial)		
Ischaemic	Myocardial infarction	IIIB
	LV dilatation/dysfunction	I
Non-ischaemic	LV dilatation/dysfunction	I
	Left atrium dilatation	I

LV, left ventricle.

**Table 2:** Causes of aortic regurgitation

Primary (valve apparatus)		
Congenital	Bicuspid, quadricuspid	
	Ventricular septal defect	
Myxomatous	Fibroelastic deficiency	
Degenerative	Age-related	IIIB
	Mucopolysaccharidoses	IIIA
Inflammatory	Rheumatic	IIIA
	Radiation (lymphoma, breast)	IIIA
	Drugs (dopamine agonists)	IIIA
	Infective endocarditis	IB
	Collagen vascular diseases	
Secondary (aortic)		
Congenital	Annuloaortic ectasia	I
	Loezs-Dietz, Marfan, Ehlers-Danloss	I
Acquired	Hypertension	I
	Atherosclerotic	I
	Infection (syphilis)	I
	Inflammation (Takayasu)	I

Identifying structural abnormalities leading to MR requires imaging of the leaflets, chordae, papillary muscles and annulus at high temporal and spatial resolution. 2D TTE is the main method to define leaflet thickening (use fundamental imaging), leaflet mobility (normal, increased (including prolapse) or restricted), degree of leaflet coaptation, as well as extent and location of calcification (none, mild, moderate and severe). 3D TTE is of incremental value in identifying the lesion, leaflet involved and the specific valve segments affected, with an accuracy similar to multiplane 2D TOE.^9^ 3D TOE has greatest accuracy overall compared with surgical findings, especially in identifying flail segments and the location and extent of prolapsed segments. While 2D TTE can diagnose the apical displacement of the mitral coaptation point, restriction in mobility and asymmetric tethering that are typical findings in ischaemic MR, 3D TTE and TOE quantify the extent of tethering that can direct surgical strategies with greater accuracy.^10^ Cardiovascular magnetic resonance (CMR) can also identify leaflet (including segmental) anatomy and motion using 2D cine imaging, although true 3D imaging is not available. It is best used when echocardiography is unable to image the valve adequately.

Visualisation of primary and secondary chordae requires good quality 2D TTE imaging in a modified apical two-chamber view to define location, length and thickening, with the transgastric long-axis view on 2D TOE also useful to perform these measurements and define interpapillary muscle distance. 3D TOE can improve on these measures but is mainly used to measure annulus size and detect annular remodelling that can be specific to a disease process, for example, to myxomatous or ischaemic MR.^11^ CMR can accurately locate, measure size and track papillary muscle displacement, but the slice thickness (5–7 mm) means that it is less useful for imaging the chordae. CMR can also identify associated fibrosis or infarction (in either the myocardium or papillary muscles),^12^ which can inform the assessment of the mechanism of MR and likelihood of improvement. Contrast-enhanced multislice CT can measure mitral annular size with similar accuracy and reproducibility to 3D TOE and has the advantage of accurate localisation and quantification of calcification.^13^ As a result, CT has a growing role in preprocedural imaging for percutaneous mitral intervention but does not yet play the central role it performs in assessment for transcatheter aortic intervention. It can also assess leaflet motion with full cardiac cycle imaging, although the temporal resolution is much lower than echocardiography or CMR, and the radiation dose is significantly higher, so this is generally reserved for rare situations when neither echocardiography nor CMR is able to provide functional information.

In the anatomical assessment of AR, 2D (and to some extent 3D) TTE remain the first step in the assessment of leaflet mobility, thickening and calcification required to determine mechanism and aetiology. When TTE provides inadequate visualisation of structures, or when high spatial and temporal resolution imaging is required to assess suitability for intervention, for example, with highly eccentric jets and when repair is considered, 2D and 3D TOEs provide incremental accuracy.^14^ Since many patients presenting with severe AR have aortic root or supracoronary ascending aortic enlargement,^7^ an additional requirement is for accurate multiplanar imaging of the aortic root and ascending aorta. Although TTE, TOE and CMR can offer this, multidetector CT provides fast, high resolution imaging of the aortic root and thoracic aorta without limitation by acoustic window and is the technique of choice, not least due to the capacity to locate and quantify calcification.^15^

## Severity of regurgitation

When quantifying severity of a regurgitant lesion, it is important to consider the appearance of (1) the valve morphology and (2) the size, shape and function of the ventricle. For example, if the mitral valve leaflets are flail, it is highly likely there is severe regurgitation but if you do not think it is severe, make sure you have done all you can to be certain. Likewise, if the LV is dilated with spherical remodelling, if you are going to call any AR moderate, make sure you have done all you can to be certain.

Good quality TTE with Doppler can provide all the core information required for evaluation of regurgitation severity but requires an experienced, motivated echocardiographer able to perform the necessary integrated assessment of multiple qualitative and quantitative parameters, in the knowledge that single measures are subject to variability^16^ (table 3). A practical problem is that there is over-reliance on colour Doppler, particularly if limited to visualisation of the regurgitant jet area, which is inaccurate (even if indexed to chamber area).^17^ Despite guidelines recommending that regurgitation is quantified using methods including vena contracta and flow convergence on colour Doppler, many decisions regarding severity of MR are still made on echo by visually estimating severity based on the size of the colour jet, with some including semiquantitative measures (vena contracta width, regurgitant orifice area), but only a minority of reports provide RVols and fraction.^18^ Flow convergence, better known as the proximal isovelocity surface area (PISA) method, is probably the most widely used method used to quantify MR, which relies on measuring the radius of a hemisphere from the point of aliasing on colour Doppler to the vena contracta of the jet. The effective regurgitant orifice (EROA) is then calculated from the maximal velocity of the regurgitant jet, and the RVol by multiplying this with the velocity time integral of the MR jet.^19^ Although quantitative grading of MR is an important independent imaging marker of outcome,^20^ it involves a number of assumptions and calculation from multiple measurements, with significant variation between expert readers, and there is increasing awareness of inaccuracy where the MR does not form a true hemisphere, particularly in functional regurgitation.^21^

**Table 3:** Multiparametric assessment of regurgitation

Parameter	Advantages	Limitations	Mild	Severe
2D colour Doppler jet area	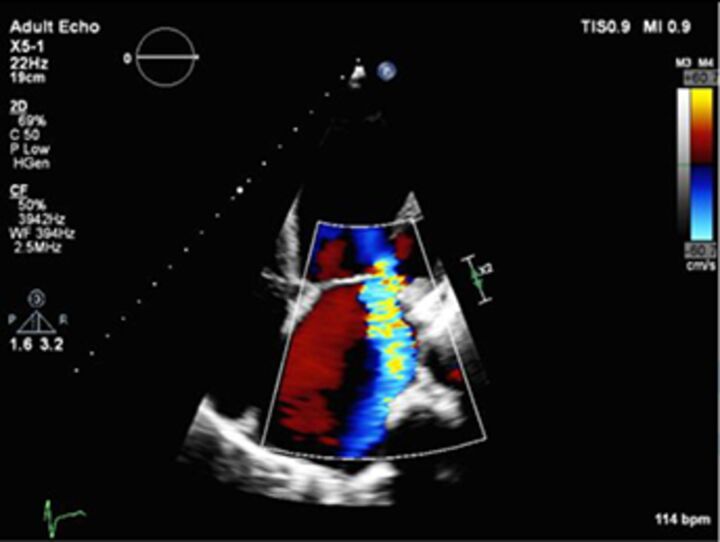	Easy screening for mild or severe If narrow, specific for mild	Subjective Overestimates if transient Underestimates if Wall hugging. Eccentric. Variable a/c to Machine settings. Haemodynamics.	Narrow origin, small	Wide origin, large MR >50% MR/LA area AR>65% jet width/LVOT
2D colour Doppler vena contracta width	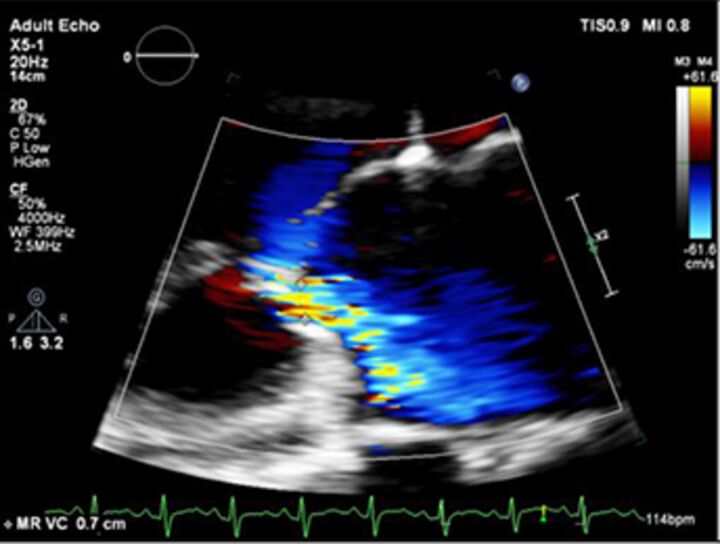	Easy Good for mild or severe Independent of flow and pressure Useful in eccentric jets Marker for ROA	Multiple jets Overestimates if transient Must be measured when US is perpendicular	<3 mm	MR >7 mm AR >6 mm
2D colour Doppler flow convergence	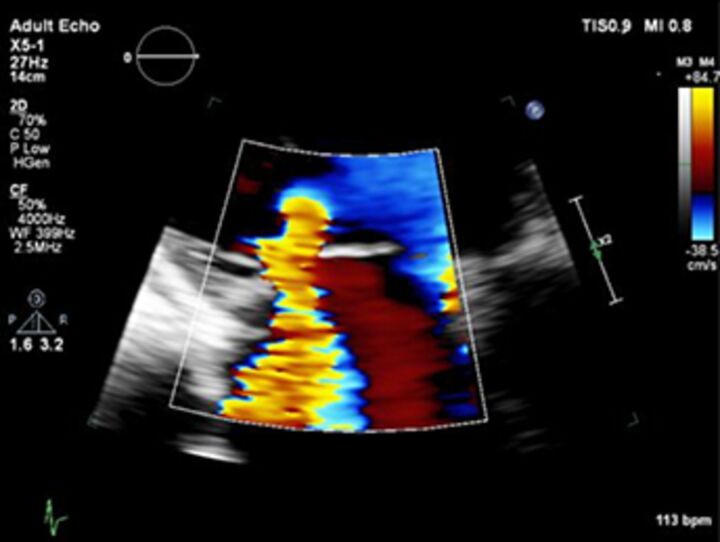	Easy If absent, specific for mild	Multiple jets Overestimates if transient. Non-hemispheric. Underestimates if Wall hugging. Eccentric.	If no flow convergence can be seen	>10 mm if Nyquist 30–40 cm/s
3D colour Doppler vena contracta area	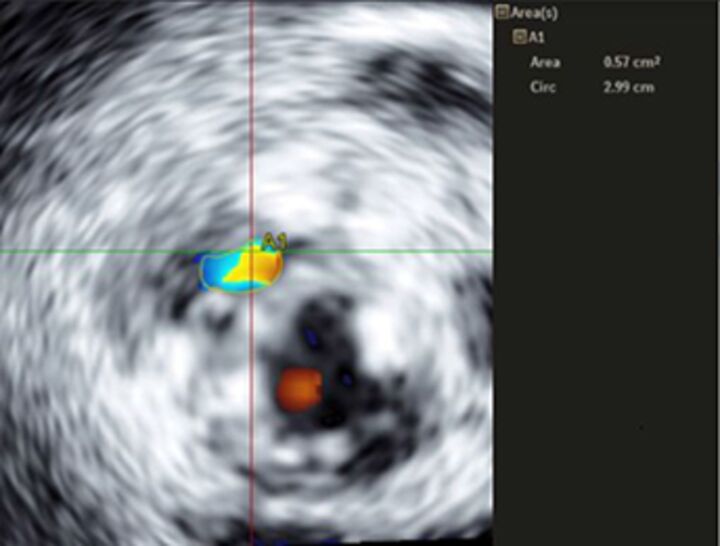	Useful if: Multiple jets If PISA non-hemispheric	Limits of temporal and spatial resolution 3D CF Overestimates if transient Slow		>40mm2
CW Doppler density	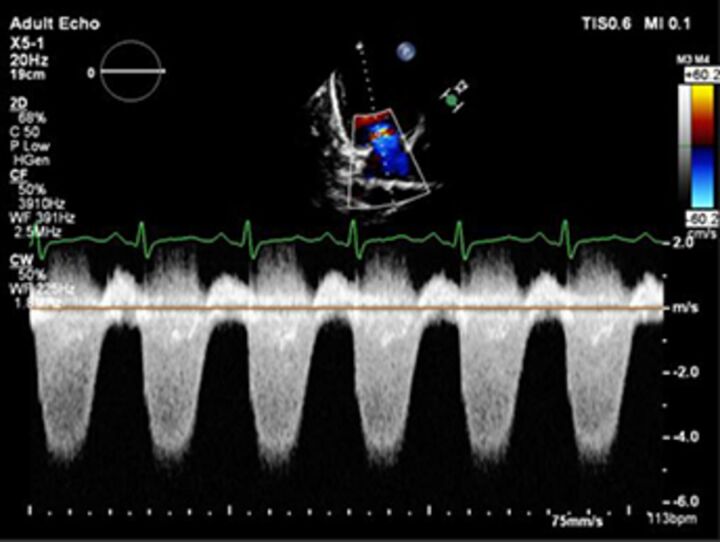	Easy Dense triangular shape specific for severe MR	Subjective Gain dependent Underestimates eccentric	Feint	Dense
2D colour Doppler PISA	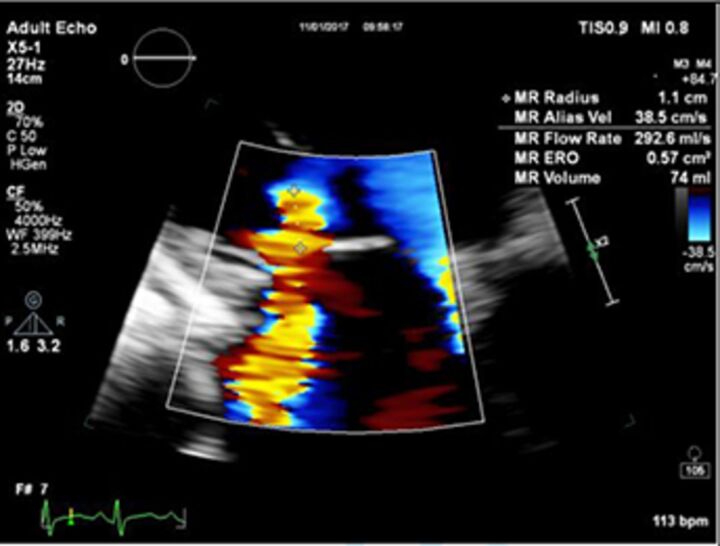	Quantitative: EROA mm2 RVol mls RF % Prognostic	Overestimates if Eccentric Transient Less accurate in functional MR	MR EROA<20 mm AR EROA<10 mm RVol<30 mL RF<30%	Primary MR>40 mm SIMR>20 mm AR EROA>30 mm RVol>60 mL RF>50%
Pulse Doppler	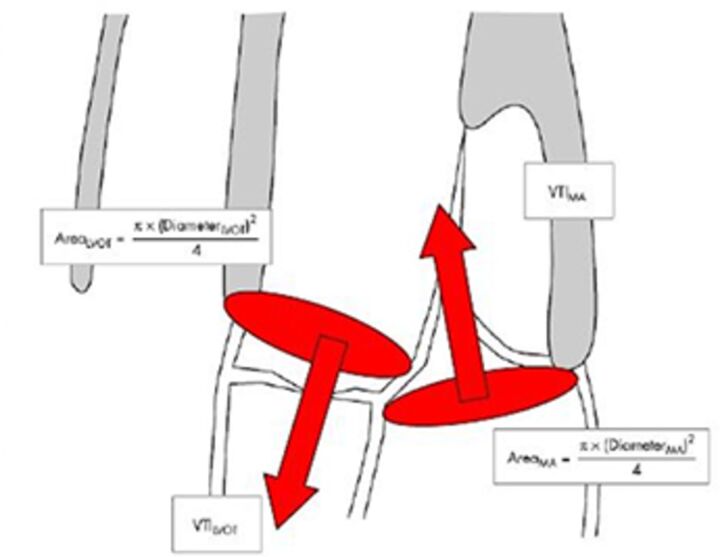	Quantitative: RVol mL RF % Can be used in Eccentric Transient Multiple jets	Wide confidence limits Difficult Not useful if multiple valve disease	Primary MR RVol <30 mL MR RF <30% AR RVol <30 mL AR RF <30%	Primary MR RVol >60 mL MR RF >50% Secondary MR RVol >30 mL AR RVol >60 mL AR RF >50%
CMR flow quantification	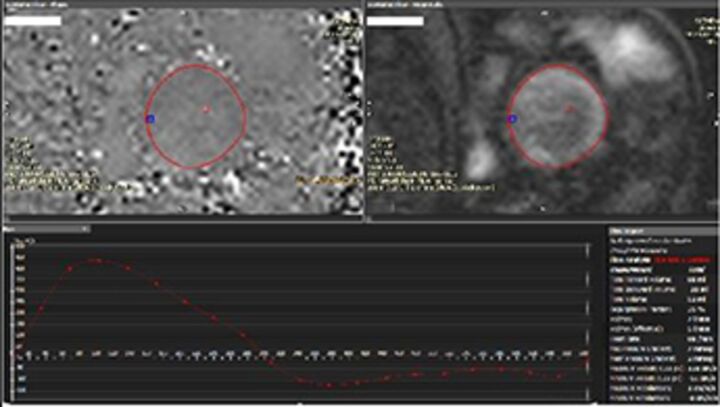	Accurate quantification of flow Not affected by irregular, eccentric, multiple or dynamic jets Easy to measure	Accurate measures often require good correction of background flow offset error Slight tendency for AR quantities to be underestimated Not available in all hospitals	MR Vol <30 mL MR RF <20% AR RF <15%	MR Vol>60 mL MR RF >40% AR RF >35%–40% Holodiastolic descending aorta flow reversal
MR only					
Pulse Doppler MV inflow	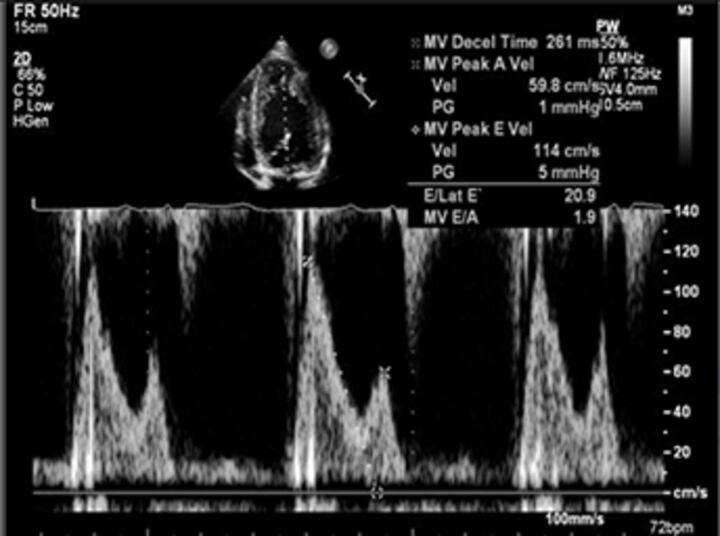	Easy Quantitative	AF Altered by LA/LV pressure gradient	A wave dominant Specific for mild MR	E Vmax >1.5 m/s
Pulse Doppler MV inflow/LVOT vti ratio	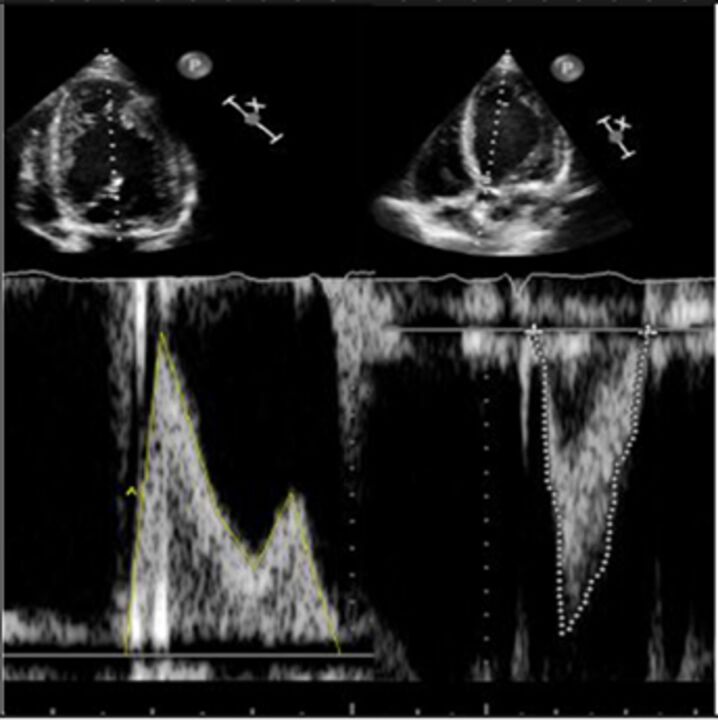	Easy	AF Not useful if multiple valve disease	<1	>1.4
Pulse Doppler pulmonary venous flow	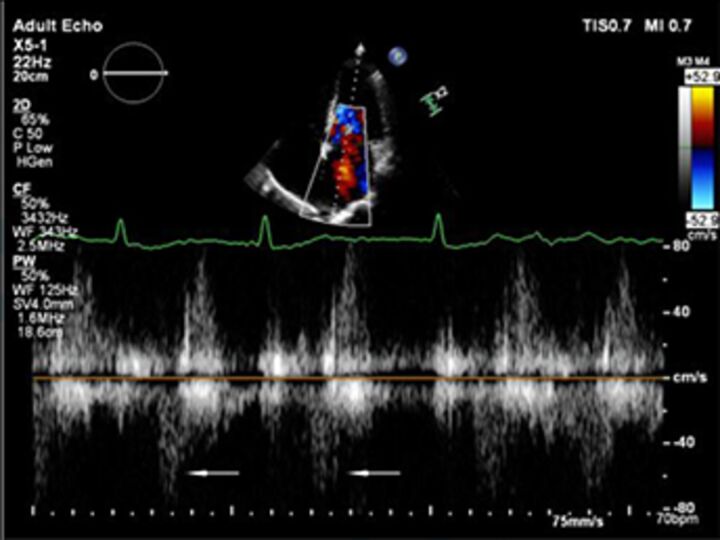	Easy on TOE Useful in eccentric or multiple jets	Systolic blunting occurs if high LA or LV end-diastolic pressure Other causes for high E Vmax	Systolic flow dominant	Systolic flow reversal
AR only					
Pulse Doppler descending aortic flow	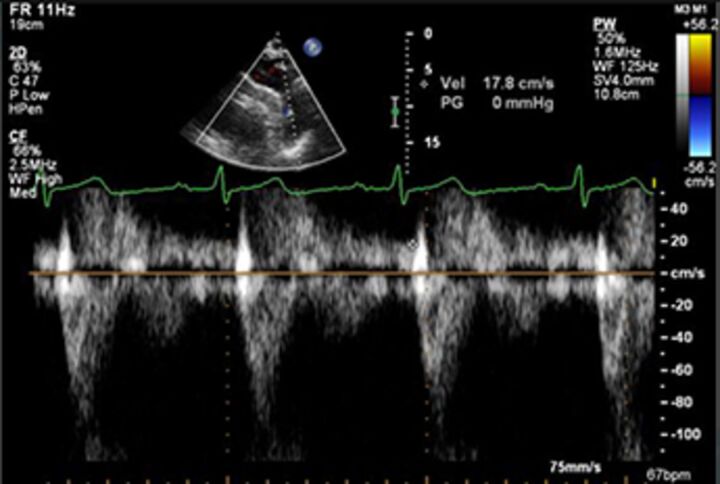	Easy If present in abdominal aorta, specific for severe AR	Less reliable when aortic stiffness increased (age) Depends on alignment of Doppler with jet direction Variable according to location of sample	Brief flow reversal is normal	Holodiastolic, end-diastolic flow Vmax>20 cm/s
CW Doppler pressure half-time	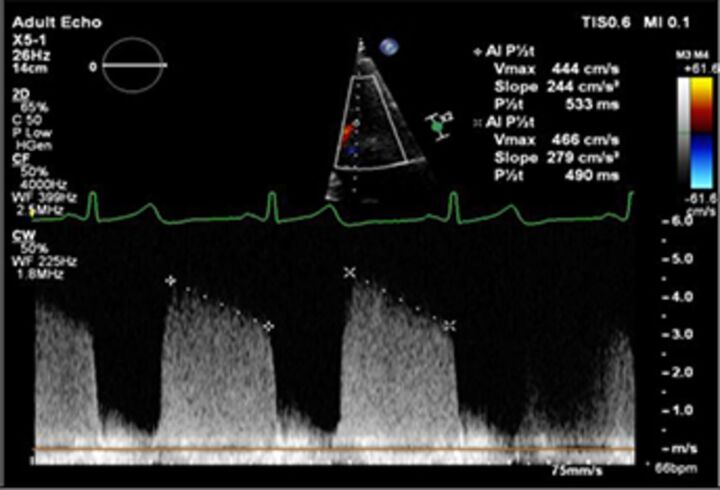	Easy If long, specific for mild	Affected by any factor altering aortic-LV pressure gradient Depends on alignment of Doppler with jet direction	>500 ms	<200 ms

a/c, according to; AR, aortic regurgitation; CF, colour flow; CMR, cardiovascular magnetic resonance; EROA, effective regurgitant orifice; LA, left atrium; LV, left ventricle; LVOT, left ventricular outflow tract; MR, mitral regurgitation; MV, mitral valve; PISA, proximal isovelocity surface area; RF, regurgitant fraction; RVol, regurgitant volume; TOE, transoesophageal echocardiography; US, ultrasound.

The assessment of AR severity with echocardiography is more challenging than that of MR. On TTE, the AR jet is seen with the highest resolution in the parasternal long-axis view, which can be used to measure vena contracta and jet width relative to the left ventricular outflow tract. In this view however, the PISA in AR is perpendicular to the angle of insonation (and therefore unreliable), and in the apical window it is in the far field and is often too small to measure reliably. In addition, the pulse Doppler method used to calculate stroke volumes across the mitral valve and left ventricular outflow tract (AR RVol=SVol _LVOT_–SVol _MV_) is subject to large interobserver variability.^22^ Other methods such as pressure half time of the AR Doppler slope can be helpful to identify mild or severe disease but may be unreliable when there are changes in LV compliance or use of antihypertensive medication, and likewise, aortic diastolic flow reversal in the descending aorta may be useful but can also occur due to changes in aortic distensibility in the elderly.

There is increasing recognition of the limitations of echocardiography in quantifying regurgitation, particularly in differentiating moderate from severe regurgitation.^23^ CMR has the advantage of direct flow quantification using phase contrast velocity mapping. When used in the aortic root, this can quantify AR Rvol directly and can also be used to quantify MR indirectly, in conjunction with LV stroke volumes (MR RVol=LV stroke volume–aortic forward flow). This has greater reproducibility than multiparametric echocardiography for RVol and grade^24^ and has good accuracy in in vitro models.^25^ Echo tends to overestimate severity compared with CMR, especially when MR varies throughout systole,^26^ and CMR is a better predictor of the future requirement for surgery, and ventricular remodelling postsurgery in the limited number of studies to date.^27 28^ Cut-offs that predict adverse events or need for intervention are therefore lower for both MR^28^ and AR,^29^ and are not always the same as guidelines based on echo parameters (see table 3). CMR is not without problems, including background phase offset error, variation in volumes due to difference in selection of the basal slice and impact of irregular heart rhythms, and is not as widely available as echocardiography. CMR is most useful for sorting out difficult cases, such as ‘moderate–severe MR’, discrepancy between symptoms and regurgitation grade, exercise capacity and severity of regurgitation on echo, and where TTE images are suboptimal.

## Assessing the consequences of regurgitation

Chronic AR is characterised by combined volume and pressure overload. The LV increases diastolic volume to accommodate AR, which results in increased diastolic wall stress. As the LV must eject a greater stroke volume over the same systolic ejection period, systolic pressure, afterload and systolic wall stress progressively increase. The combination of volume and pressure overload result in LV dilatation, eccentric LV hypertrophy, and increased interstitial fibrosis, which are progressive with increasing severity of AR and can lead ultimately to heart failure and death.^30^ In chronic MR, volume overload produces similar LV changes, with gradual transition from a compensated phase, transitioning to LV impairment and ultimately decompensation.^31^ There is some CMR evidence that AR results in higher LV volumes for the same quantity of regurgitation, suggesting a differential LV response.^32^ Current guidelines emphasise the role of tracking LV chamber size and function, with cut-offs for intervention in asymptomatic patients based on linear dimensions and ejection fraction on TTE^33^ (tables 3). There are obvious limitations to using a single linear dimension as a trigger for intervention, not least that in primary MR, there is preferential remodelling of the LV in the apex and mid cavity.^34^ Volumetric data have not however, been established in guidelines as a trigger, whether by contrast TTE, 3D TTE or CMR, despite the greater reproducibility and accuracy in tracking change. Likewise, there are limitations to the use of ejection fraction in regurgitation, which measures chamber size rather than myocardial contractility and is altered by the RVol itself.

Multiple alternatives have been proposed for early detection of myocardial dysfunction, including fall in systolic and early myocardial relaxation velocities on tissue Doppler, reduction in strain, contractile reserve on stress echocardiography, and fibrosis on CMR, none of which have been accepted into guidelines or been subjected to randomised trial end-point adjudication.^35^

Measuring left atrial volume, RV size and function, and maximal velocity of the regurgitant TR jet are all important parts of the imaging assessment of the patient with MR that carry prognostic weight, and provide supportive evidence for a decision to intervene.^16^ It is important to recognise the limitations in accuracy of the calculation of pulmonary artery systolic pressure by echocardiography however, and the need for confirmation of the estimation of pulmonary pressure by invasive testing.^36^ CMR offers greater inter-study reproducibility for volume measures of LV, RV and LA chamber size and ejection fraction, compared even to 3D TTE with or without contrast but proof of an advantage in routine assessment of the asymptomatic patient with MR or AR is lacking.^37^

## Summary

Imaging assessment of the patient with MR or AR requires detailed assessment of the valve apparatus, associated cardiac chambers, the aortic root (for AR) and flow quantification (for CMR) to identify the cause and mechanism of regurgitation, severity, and haemodynamic impact on ventricular and atrial remodelling, as well as the effect on pulmonary pressure. Transthoracic echocardiography remains the workhorse but requires careful integration of multiple parameters that vary in sensitivity and specificity, particularly when the aetiology is ischaemic, and the skill to use 3D regularly. Where data are conflicting, imaging is suboptimal or when intervention is under consideration, either transoesophageal echocardiography or CMR can be used, with 3D an integral component for complete TOE assessment.

**Key points**

- All imaging assessments in mitral or aortic regurgitation should include a statement on the quality of the scan, the size of the patient (height, weight and body surface area) and haemodynamic status (heart rate, blood pressure and procedural medication).
- Transthoracic echocardiography remains the first-line imaging modality to determine cause and mechanism of regurgitation, quantify severity using multiple parameters, and impact of regurgitation on atrial and ventricular size and function.
- In cases where the above information is incomplete or is contradictory, transoesophageal echocardiography using 2D and 3D is the usual next step for detailed assessment of valve anatomy and helps with quantification.
- In those cases where regurgitation severity and the effects of regurgitation on chamber size and function are the primary source of concern, cardiovascular magnetic resonance (CMR) may be a better, less invasive alternative.
- Grading of severity by echocardiography and CMR differ, with thresholds for intervention lower using CMR, although there is a lack of data using these to guide intervention.
- Multislice CT is currently used mainly to image specific subsets of patients before intervention, such as those for percutaneous valve intervention and those requiring concomitant aortic surgery.

**CME credits for Education in Heart**

Education in Heart articles are accredited for CME by various providers. To answer the accompanying multiple choice questions (MCQs) and obtain your credits, click on the 'Take the Test' link on the online version of the article. The MCQs are hosted on BMJ Learning. All users must complete a one-time registration on BMJ Learning and subsequently log in on every visit using their username and password to access modules and their CME record. Accreditation is only valid for 2 years from the date of publication. Printable CME certificates are available to users that achieve the minimum pass mark.

10.1136/heartjnl-2019-316216.supp1 Supplementary data
