# Supplementary material for: Imaging assessment of mitral and aortic regurgitation: current state of the art
Source: Heart. 2020 Aug 17;106(22):1769–76. doi: 10.1136/heartjnl-2019-316216 (PMC7656154; doi:10.1136/heartjnl-2019-316216)
Supplement: Supplementary data [file heartjnl-2019-316216supp001.pdf]

A. With respect to valve regurgitation:

1. The mitral regurgitant orifice area in rheumatic disease is dynamic. T

2. The aortic regurgitant orifice in degenerative disease is fixed.

3. Use of benzodiazepine sedative during transoesophageal echocardiography may increase MR.

4. Systemic hypotension increases severity of aortic regurgitation.

5. Elevated left atrial pressure increases regurgitant flow.

Explanation: Regurgitant orifices are always dynamic, even in advanced rheumatic mitral stenosis. BZD sedation usually decreases regurgitation, and systemic hypotension leads to under-estimation of AR.

B. With respect to mechanism and aetiology of regurgitation:

1. Physiological regurgitation becomes more common with age.T

2. Mild AR is often found in younger patients and is a normal variant.

3. AR in younger patients is due to a bicuspid valve in the majority.

4. In secondary MR, apical displacement of co-aptation is due to annular dilatation.

5. Leaflet thickening does not occur in ischaemic MR.

Explanation: Mild AR is rarely a normal variant at any age, and is most often due to annulo-aortic dilatation. Apical displacement of co-aptation is due to papillary muscle separation or traction on chordae due to displacement. Recent data highlights that leaflets thicken to compensate for annular dilatation.

C. In imaging the mitral valve apparatus:

1. Cardiovascular magnetic resonance imaging has the same ability to image the chordal attachments as echocardiography.

2. CT provides equivalent mitral annular dimensions to 2D echocardiography.

3. Trans gastric 2D transoesophageal echocardiography remains gold standard for chordal assessment.T

4. Quantification of papillary muscle and inferolateral fibrosis is important in determining surgical outcomes in mitral valve prolapse.

5. Localisation of calcification in the mitral annulus is irrelevant when surgical replacement is planned.

Explanation: CMR does many things as well as echo but not things that require high spatial resolution such as chordae. CT does not provide equivalent annular dimensions to 2D echo, since the latter is frequently wrong and cannot measure any of the true annular lengths from standard planes, without 3D echo MPR. There are no data relating fibrosis in MR to outcomes, while localisation of posterior annular calcification is critical to planning surgical approaches.

D. When quantifying severity of mitral regurgitation:

1. Indexing jet size on colour Doppler to atrial size is a reliable method for assessment of MR severity.

2. E wave dominance in mitral inflow is a reliable sign of non-severe MR.

3. Quantification of regurgitation by echocardiography over-estimates severity compared to CMR.T

4. Systolic pulmonary artery pressure is a trigger for intervention in primary MR, and measurement on transthoracic echocardiography is accurate.

5. Pulmonary venous inflow is a reliable method of quantifying secondary MR.

Explanation: Jet size whether indexed or not is unreliable, E waves get bigger with more severe MR, and measurement of PASP on echocardiography is frequently inaccurate due to the poor accuracy of estimation of RA pressure based on IVC dimension, among other factors. Pulmonary venous inflow is affected by high LVEDP so is often not accurate in secondary MR.

E. When quantifying severity of aortic regurgitation:

1. PISA methods are often useful for quantifying AR on transthoracic echocardiography.

2. Flow reversal in the proximal descending aorta is more accurate with older age.

3. Accuracy of CMR phase velocity mapping does not vary according to the jet eccentricity or regurgitant orifice shape.T

4. Echocardiographic measurement of flow in the LVOT and mitral or pulmonary stroke volume by pulsed Doppler is highly reproducible for calculation of AR severity.

5. Summation of vena contracta is useful when multiple AR jets are present, eg in perforation.

Explanation: PISA is often difficult to obtain on TTE in AR, flow reversal gets less accurate due to the effect of aortic stiffness, while pulse Doppler methods that are often central to guidelines are not often used in practice but more importantly lack repeatability. Vena contracta cannot be summated, unless measured by 3D as vena contracta area.

F. In tracking ventricular size and function over time:

1. Use of volumes and ejection fraction are mandated by guidelines as cut-offs for intervention in regurgitation.
2. Basal linear dimensions are accurate in measuring ventricular remodelling in MR.
3. Quantifying left atrial volume is unhelpful in predicting prognosis in MR.
4. Haemodynamic status can affect measurements in MR.T
5. Selection of the basal slice for CMR quantification of ventricular volumes does not affect inter-observer variability for end-diastolic volume or ejection fraction.

Explanation: Linear dimensions are still required by guidelines as indications for intervention, and basal linear dimensions do not reflect the remodelling that occurs in MR. LA volume is an important predictor of outcome in MR and selection of the basal slice is a notorious practical variable in measuring volumes in CMR.
